# Maternal Height and Preterm Birth: A Study on 192,432 Swedish Women

**DOI:** 10.1371/journal.pone.0154304

**Published:** 2016-04-21

**Authors:** José G. B. Derraik, Maria Lundgren, Wayne S. Cutfield, Fredrik Ahlsson

**Affiliations:** 1 Liggins Institute, University of Auckland, Auckland, New Zealand; 2 Department of Women's and Children's Health, Uppsala University, Uppsala, Sweden; Hospital de Especialidades del Niño y la Mujer de Queretaro, MEXICO

## Abstract

**Background:**

There is increasing evidence that lower maternal stature is associated with shorter gestational length in the offspring. We examined the association between maternal height and the likelihood of delivering preterm babies in a large and homogeneous cohort of Swedish women.

**Methods:**

This study covers antenatal data from the Swedish Medical Birth Register on 192,432 women (aged 26.0 years on average) born at term, from singleton pregnancies, and of Nordic ethnicity. Continuous associations between women's heights and the likelihood of preterm birth in the offspring were evaluated. Stratified analyses were also carried out, separating women into different height categories.

**Results:**

Every cm decrease in maternal stature was associated with 0.2 days shortening of gestational age in the offspring (p<0.0001) and increasing odds of having a child born preterm (OR 1.03), very preterm (OR 1.03), or extremely preterm (OR 1.04). Besides, odds of all categories of preterm birth were highest among the shortest women but lowest among the tallest mothers. Specifically, women of short stature (≤155 cm or ≤-2.0 SDS below the population mean) had greater odds of having preterm (OR 1.65) or very preterm (OR 1.47) infants than women of average stature (-0.5 to 0.5 SDS). When compared to women of tall stature (≥179 cm), mothers of short stature had even greater odds of giving birth to preterm (OR 2.07) or very preterm (OR 2.16) infants.

**Conclusions:**

Among Swedish women, decreasing height was associated with a progressive increase in the odds of having an infant born preterm. Maternal short stature is a likely contributing factor to idiopathic preterm births worldwide, possibly due to maternal anatomical constraints.

## Introduction

Preterm birth is not only a major cause of neonatal mortality worldwide, but it is also associated with adverse short- and long-term health outcomes [1]. Approximately half of all preterm births are idiopathic or spontaneous, whose causes are multifactorial and often unknown [1,2]. However, maternal height may be a contributing factor. There is increasing evidence that lower maternal stature is associated with shorter gestational length in the offspring [3–6], including evidence from a systematic review and meta-analysis [7]. As a result, short mothers are more likely to deliver preterm babies [4,5,7].

It is not easy to dissect the influence of maternal height on length of gestation, since the latter may be affected by a number of factors such as ethnicity [2], maternal gestational age [8], and maternal obesity [9]. Thus, in this study we examined the association between maternal height and the likelihood of delivering preterm babies in a large and relatively homogeneous cohort of Swedish women.

## Methods

We examined Swedish Medical Birth Register data, from the first antenatal visit on all singleton Swedish women aged ≥18 years who were born at term (37–41 weeks of gestation) and gave birth in 1991–2009. For women with two or more pregnancies in the study period, data were only included for the first recorded pregnancy. Women were excluded if they were of non-Nordic ethnicity, of very short stature (≤130 cm), born outside the term range or small-for-gestational-age [SGA; <-2 standard deviation scores (SDS) in birth length or birth weight], or born with congenital malformations (ICD-9 740–759 and ICD-10 Q0–Q99). The occurrence of a previous preterm pregnancy was not accounted for. Height was recorded to the nearest cm, but it was self-reported in some cases. Gestational age of the women at their birth was mostly estimated from the date of the last menstrual period, otherwise estimates were based on ultrasound scans.

Relevant data (i.e. gestational age and birth weight) were also obtained on all offspring (with no exclusions) from the Birth Register. Offspring gestational age was based on ultrasound examination in the second trimester.

A continuous association between maternal height and gestational age in the offspring was evaluated using general linear regression models, adjusting for maternal factors (age, maternal gestational age, BMI, and smoking during pregnancy), as well as infant sex. Logistic regression models (adjusting for above confounders) were run to evaluate likelihood of having an infant born preterm (<37 weeks of gestation), very preterm (<32 weeks of gestation), or extremely preterm (<28 weeks of gestation). Stratified analyses were also carried out, dividing the women into groups according to height SDS based on the study population mean. In particular, short stature (≤155 cm) was defined as height ≤-2 SDS (below the study population mean) and tall stature as ≥2 SDS (≥179 cm). Analyses were performed in SAS v.9.4 (SAS Institute, Cary, USA). Where applicable, results are expressed as odds ratios (OR) or ß coefficients, with associated 95% confidence intervals (CI).

This study was approved by the Regional Ethical Review Board in Uppsala.

## Results

There were 303,301 women born in 1973–1988 in Sweden who gave birth in 1991–2009. Anthropometric data were available on 268,208 women, but 75,776 failed to meet inclusion criteria (i.e. women born outside the term range, SGA or with malformation, aged <18 years, being of non-Nordic ethnicity, or born from multiple pregnancies). Thus, this study covers data on 192,432 women and their newborns, whose demographic characteristics are shown in Table 1.

**Table 1:** Characteristics of the study population of 192,432 Swedish women and their newborns.

**Women**	Age	26.0 ± 3.9
	Height (cm)	167.2 ± 5.9
	Weight (kg)	67.4 ± 12.8
	BMI (kg/m^2^)	24.1 ± 4.3
	Smoking during pregnancy	11.4%
**Newborns**	Sex ratio (males)	51.4%
	Birth weight (kg)	3.49 ± 0.55
	SGA	1.9%
	Gestational age (weeks)	39.4 ± 1.9
	Preterm (<37 weeks of gestation)	6.0%
	Very preterm (<32 weeks of gestation)	0.7%
	Extremely preterm (<28 weeks of gestation)	0.2%

Where appropriate, data are means ± standard deviations.

Decreasing maternal stature was associated with lower gestational age in the offspring [ß = 0.022 (95% CI 0.020, 0.023); p<0.0001], so that every cm decrease in maternal height was associated with 0.2 days shortening of pregnancy length. Thus, the shorter the mother was (in cm), the greater were the odds of having a child born preterm (OR 1.03; 95% CI 1.03, 1.03), very preterm (OR 1.03; 95% CI 1.02, 1.04), or extremely preterm (OR 1.04; 95% CI 1.02, 1.06).

**Fig 1:**
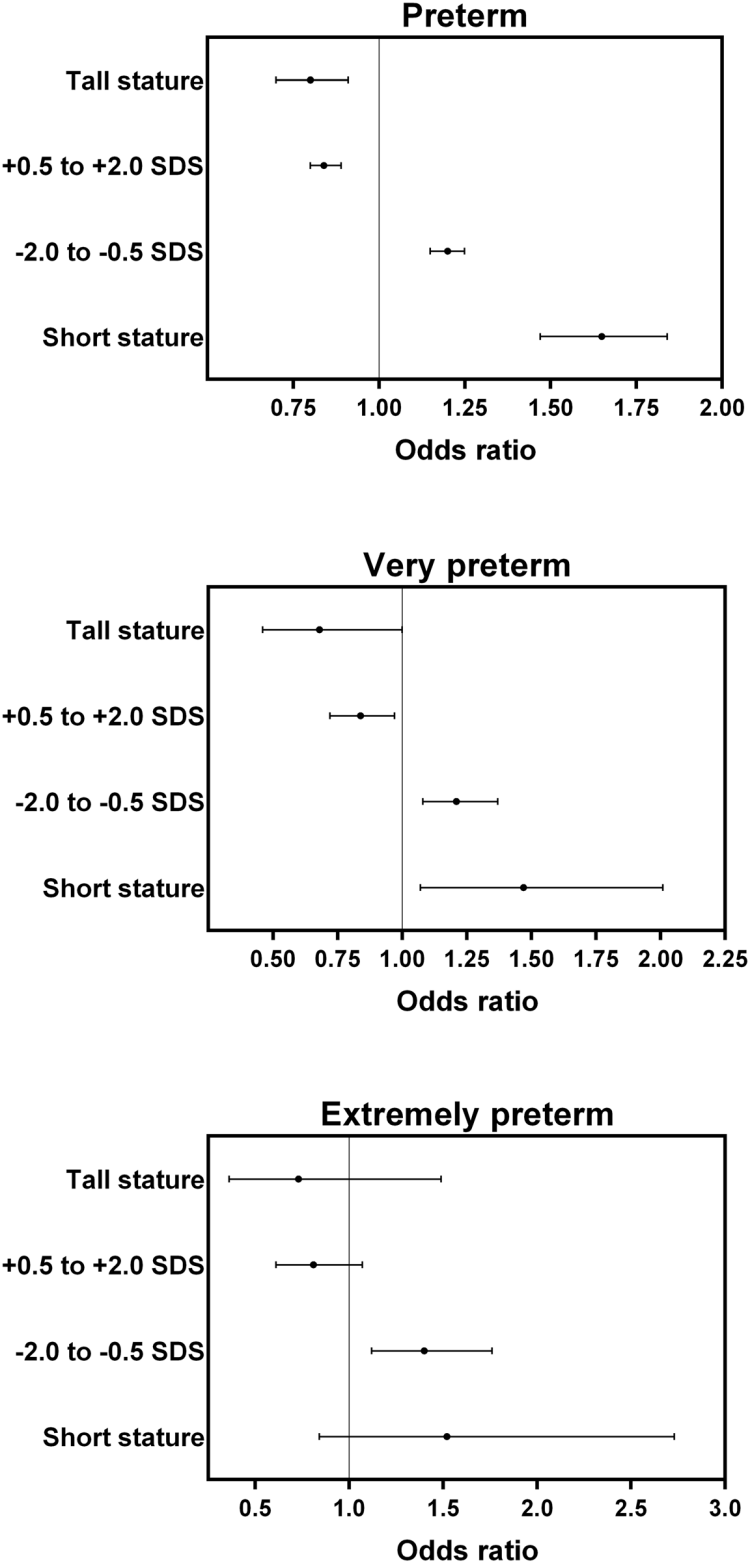
Maternal height categories among 192,432 Swedish women and the odds of having an infant born preterm (<37 weeks of gestation), very preterm (<32 weeks of gestation), or extremely preterm (<28 weeks of gestation). Data are odds ratios and 95% confidence intervals, adjusted for maternal factors (age, maternal gestational age, BMI, and smoking during pregnancy), as well as fetal sex. The reference group are mothers with heights between -0.5 and +0.5 standard deviation scores (SDS) around the study population mean. Short stature indicates height ≤-2.0 SDS, and tall stature height ≥2.0 SDS.

Stratified analyses showed a progressive decline in the prevalence of infants born preterm, very preterm, or extremely preterm with increasing stature across height groups (Table 2). Thus, odds of any form of preterm birth were highest for the shortest women but lowest among the tallest women (Fig 1).

**Table 2:** Maternal height in Swedish women and the prevalence of infants born preterm (<37 weeks of gestation), very preterm (<32 weeks of gestation), or extremely preterm (<28 weeks of gestation).

	≤-2.0 SDS (Short stature)	-2.0 to -0.5 SDS	-0.5 to 0.5 SDS	0.5 to 2.0 SDS	≥2.0 SDS (Tall stature)
N	3,992	59,619	75,655	47,542	5,624
Proportion of the population (%)	2.1	31.0	39.3	24.7	2.9
Maternal height range (cm)	132–155	156–164	165–170	171–178	179–196
Offspring gestational age (weeks)	39.0 ± 2.2	39.3 ± 2.0	39.4 ± 1.9	39.5 ± 1.8	39.6 ± 1.8
Preterm infant (%)	9.42	6.91	5.82	4.93	4.68
Very preterm infant (%)	1.13	0.88	0.72	0.60	0.48
Extremely preterm infant (%)	0.33	0.28	0.20	0.16	0.14

SDS is standard deviation score based on the study population mean. Gestational age data are means ± standard deviations.

Specifically, women with short stature had greater odds of preterm (OR 1.65; 95% CI 1.47, 1.84) or very preterm (OR 1.47; 95% CI 1.07, 2.01) births than women of average stature (-0.5 to 0.5 SDS) (Fig 1). When compared to women of tall stature, mothers of short stature had even greater odds of giving birth to preterm (OR 2.07; 95% CI 1.76, 2.44) or very preterm (OR 2.16; 95% CI 1.33, 3.50) infants.

## Discussion

Among Swedish women, decreasing height was associated with a progressive increase in the odds of having an infant born preterm. As a result, the odds of having a child born preterm or very preterm were highest for women of short stature but lowest among the tallest women.

One of the strengths of this study was the fact that height was assessed in women within a relatively narrow age range, so that the possible confounding effects of height reduction with age [10] were minimized. In addition, we studied a relatively homogeneous population (e.g. all Nordic women born at term), which allowed us to more accurately evaluate the associations between maternal anthropometry and birth outcomes. However, as Swedish women are relatively tall, our findings cannot be readily extrapolated to other female populations of much shorter average height, such as those in Latin American or Asia [11]. Nonetheless, the same pattern of a progressive increase in odds of preterm births with decreasing stature has been observed amongst women from Asia, Latin America, Africa, and Europe [4,12,13]. A study in New Zealand (87% Caucasian women) also observed that the risk of spontaneous preterm birth after pre-labour rupture of membranes decreased by 7% for every cm increase in maternal height [14]. The authors noted however, that no association was observed between maternal height and spontaneous preterm birth with intact membranes. This level of detail could not be examined in our cohort as we did not have data on the aetiology of individual preterm births, which is the main limitation of our study. Nonetheless, our results and those from previous studies [4,12,14,15] indicate that the risk of having an infant born preterm is greatest in the shortest women within any given population or ethnicity. Further, another large study using data from the Swedish Birth Register showed that short mothers (<160 cm) had substantially increased risk of having an SGA infant [16].

Ozaltin et al. previously discussed possible mechanisms underpinning the association between maternal height and adverse outcomes in the offspring, including socioeconomic factors, under-nutrition *in utero*, and maternal anatomical constraints [17]. Although socioeconomic conditions and under-nutrition could be responsible in part for observed associations in low- to middle-income countries, they would be unlikely to be an important factor in the comparatively well-resourced Swedish population (even though there were no socioeconomic data collected on the women, which is a limitation of our study). Patel et al. speculated that the shorter gestational length in certain ethnic groups may be an evolutionary adaptation to smaller maternal pelvic size [18]. It has also been suggested that women of lower stature would have a shorter cervical length, but one study suggested that this is not the case [19]. Unfortunately, we did not have data on cervical length for our study population in order to address this question. Nonetheless, in a recent study using data on genome-wide single nucleotide polymorphism from Finland, Norway, and Denmark, Zhang et al. concluded that "the effect of maternal height on gestational age was transmitted through phenotypic causal mechanism rather than genetic inheritance" [13]. As a result, the evidence suggests that anatomical constraints are more likely to explain the effects of maternal height on pregnancy length. Further support for the anatomical explanation was the lack of effect of paternal height on gestational length that we [3] and others have observed [5]. However, it is important to note that the anatomical constraints hypothesis would likely apply only to spontaneous or idiopathic preterm deliveries.

Another limitation of this study was that height for a minority but unknown number of women was self-reported and not measured. It seems that adult women tend to overestimate their own height [20], with a Swedish study on older adults (mean age 63.9 years) indicating that height was overestimated by 0.9 to 1.2 cm [21]. Thus, it is possible that the effects of short stature on pregnancy length might have been slightly under-estimated in our study. In addition, the association of maternal stature with the risk of preterm delivery was not adjusted for other conditions associated with a higher risk of prematurity, such as polyhydramnios, short cervical length, cervical conization, and cervical incompetence. Lastly, there is a margin of error associated with the estimation of gestational length by ultrasound scans or the last menstrual period [22], so that some infants and mothers would likely have been misclassified into the wrong gestational age category. However, the possible effects of such misclassifications would likely have been minimized in our large cohort.

In conclusion, our study on a large and homogeneous cohort of Swedish women provides strong evidence of a progressive association between decreasing maternal height and increased likelihood of having a child born preterm. These data corroborate the evidence from a number of previous studies [3–5,12–15] and suggest that maternal short stature may be a contributing factor to a number of spontaneous preterm births worldwide [4]. Based on this accumulating evidence, maternal height is one of the factors that need to be considered when evaluating a woman's risk of preterm delivery.
